# Human Serum Albumin Cys^34^ Oxidative Modifications following Infiltration in the Carotid Atherosclerotic Plaque

**DOI:** 10.1155/2014/690953

**Published:** 2014-03-06

**Authors:** Antonio Junior Lepedda, Angelo Zinellu, Gabriele Nieddu, Pierina De Muro, Ciriaco Carru, Rita Spirito, Anna Guarino, Franco Piredda, Marilena Formato

**Affiliations:** ^1^Dipartimento di Scienze Biomediche, University of Sassari, Via Muroni 25, 07100 Sassari, Italy; ^2^Centro Cardiologico “F. Monzino”, IRCCS, 20138 Milano, Italy; ^3^Servizio di Chirurgia Vascolare, Clinica Chirurgica Generale, University of Sassari, 07100 Sassari, Italy

## Abstract

*Objectives.* To evaluate if the prooxidant environment present in atherosclerotic plaque may oxidatively modify filtered albumin. *Methods.* Fluorescein-5-maleimide labelled plasma samples and plaque extracts from 27 patients who had undergone carotid endarterectomy were analysed through nonreducing SDS-PAGE for albumin-Cys^34^ oxidation. Furthermore, degree and pattern of S-thiolation in both circulating and plaque-filtered albumin were assayed. *Results.* Albumin filtered in the atherosclerotic plaque showed higher levels of Cys^34^ oxidative modifications than the corresponding circulating form as well as different patterns of S-thiolation. *Conclusions.* Data indicate that the circulating albumin, once filtered in plaque, undergoes Cys^34^ oxidative modifications and demonstrate for the first time that albumin is a homocysteine and cysteinylglycine vehicle inside the plaque environment.

## 1. Introduction

Human serum albumin (HSA) is the most abundant multifunctional plasma protein (about 60% of total protein content). It is a small globular protein of 66,438 Da that accounts for both antioxidant functions such as ROS/RNS scavenging, extracellular redox balance, and redox active transition metal ion binding and transport functions for many molecules such as fatty acids, nitric oxide, hemin and drugs [1, 2]. Paradoxically, for cycling transition metal ions such as iron and copper from less reactive (ferric/cupric) to more prooxidant (ferrous/cuprious) states, albumin can also display prooxidant properties [3]. Furthermore, albumin acts as a strong inhibitor of apoptosis in cultured macrophages, neutrophils, lymphocytes, and endothelial cells [4–7]. In its primary structure, it contains 34 cysteine residues that contribute with 17 disulfide bridges to overall tertiary structure and one redox active free cysteine residue (Cys^34^), responsible for many functions described above [1, 2]. It has been reported that this highly reactive residue, which accounts for 80% (500 *μ*mol/L) of total thiols in plasma, is the preferential plasma scavenger of oxygen and nitrogen reactive species, having an unexpectedly low pKa compared to that of cysteine and glutathione [8].

HSA is present primarily in the reduced form (mercaptalbumin), although about 30–40% could be variably oxidized (nonmercaptalbumin), both reversibly as mixed disulfide with low molecular weight thiols [9], S-nitroso Cys [10], or sulfenic acid and irreversibly as sulfinic or sulfonic acid [11]. Furthermore, recently, it has been described that albumin, through nucleophilic residues and in particular Cys^34^, is the main plasma target of reactive carbonyl species such as 4-hydroxy-trans-2-nonenal, therefore acting as an endogenous detoxifying agent for these proatherogenic species [12].

Although a large number of clinical studies have associated both the albumin levels and the oxidation state of Cys^34^ to various clinical conditions such as aging [13], renal disease [14], hepatic disease [15], diabetes [16], and coronary artery disease [17–19], little is known about its pathophysiological significance.

Albumin S-thiolation by low molecular weight (LMW) thiols, such as cysteine (Cys), homocysteine (Hcy), cysteinylglycine (Cys-Gly), glutamylcysteine (Glu-Cys), and glutathione (GSH), is the most common Cys^34^ oxidative modification. Even though high plasma levels of homocysteine are known to be an important risk factor in arterial disease, the pathophysiological processes leading to arterial injury have not been fully understood yet. It has been reported that homocysteine promotes vascular endothelial dysfunctions [20], stimulates the proliferation of vascular smooth muscle cells [21], and induces extracellular matrix remodelling through activation of latent metalloproteinases [22, 23]. In this regard, also the activation of the pro-MMP-1, -8, and -9 by S-glutathionylation, via the so-called cysteine switch mechanism, has been described [24]. One interesting hypothesis on the molecular mechanisms of homocysteine action on vascular cells was proposed by Sengupta et al. [25], who suggested that albumin could be homocysteine vehicle inside the cells by some different described endocytic pathways [26–29].

We have previously demonstrated that LDL apolipoprotein B-100 is able to bind all plasma thiols [30–32] and that human carotid atherosclerotic plaques contain all LMW thiols present in plasma but with a different distribution [33]. Recently, by means of a proteomic approach on human carotid atherosclerotic plaques, we evidenced that the majority of extracted proteins were of plasma origin (about 70% of total proteins), with albumin being the most represented, and identified a panel of proteins differentially expressed/oxidized in stable and unstable lesions [34, 35].

The aim of this work was to evaluate if the prooxidant environment present in atherosclerotic plaque could oxidatively modify the filtered albumin. In particular we analysed fluorescein-5-maleimide labelled plasma and plaque extracts by nonreducing SDS-PAGE for Cys^34^ oxidation and assayed degree and pattern of HSA S-thiolation by applying a highly sensitive quantitative method recently developed by our research group [36].

## 2. Materials and Methods

### 2.1. Sample Collection

Twenty-seven atherosclerotic plaque specimens were collected from patients undergoing carotid endarterectomy and stored at −80°C until analysis. Blood samples were collected into Vacutainer tubes containing EDTA and immediately processed. After centrifugation at 2,000 g for 10 minutes at 4°C, plasma was separated and stored at −80°C until analysis. Informed consent was obtained before enrolment. The study was approved by the local Ethical Committees of the University of Sassari and of Centro Cardiologico “F. Monzino,” IRCCS, in accordance with institution guidelines and conformed to the principles outlined in the Declaration of Helsinki.

### 2.2. Plaque Proteins Extraction

Plaque segments were thawed at 4°C, washed in phosphate buffered saline to remove residual blood, weighed, and finely minced with a tissue slicer blade. Protein extraction was conducted in a buffer containing 6 mol/L guanidinium chloride, 50 mmol/L sodium acetate, 100 *μ*mol/L 4-amidinophenylmethanesulfonyl fluoride, 2 *μ*g/mL Kallikrein inactivator, and 50 *μ*mol/L leupeptin (pH 7) at a ratio of 7 mL of extraction buffer for 1 g of wet weight tissue, under continuous shaking for 1 hour at room temperature. The resulting suspension was centrifuged at 65,000 g in a TL-100 centrifuge (Beckman Coulter, Brea, USA) for 30 minutes at 20°C. Extracts were delipidated [37] and resolubilized in 250 mmol/L Tris, 4% SDS, pH 7. Protein concentration was quantified with the DC Protein Assay Kit (Bio-Rad, Hercules, USA) using bovine serum albumin as a standard.

### 2.3. Fluoro-Tagging of Protein Reduced Sulfhydryl Groups

To evaluate HSA-Cys^34^ residue oxidation, we analysed fluorescein-5-maleimide (F5M) labelled plasma samples and plaque extracts by nonreducing SDS-PAGE, followed by fluorescence image acquisition and Coomassie Brilliant Blue G250 staining [35]. A calibration curve with commercial bovine serum albumin, ranging from 0.04 to 1.0 *μ*g, was set up. Both standards and samples were incubated with phosphate buffered saline containing 25-fold molar excess of F5M for two hours in the dark at room temperature following the manufacturer instructions (PIERCE Biotechnology, Rockford, USA). The fluorescent probe used is known to be effective for labelling reduced protein sulfhydryl groups at pH 6.5–7.5 forming a stable thioether bond [38].

### 2.4. Nonreducing SDS-PAGE

After F5M labelling both standards and samples were solubilised with Laemmli buffer 4X containing 250 mmol/L Tris, 8% SDS, 40% glycerol, 0.0008% bromophenol blue, and pH 6.8 at 60°C for 30 minutes. 4 *μ*L of each of derivatized standards and samples (about 1 *μ*g and 20 *μ*g of total proteins for plasma and plaque extracts, resp.), in duplicate, was resolved by Tris-glycine SDS-PAGE in 0.75 mm thick 10% T, 3% C running gel with a 5% T, 3% C stacking gel, in a Mini-Protean Tetra cell vertical slab gel electrophoresis apparatus (Bio-Rad, Hercules, USA). Electrophoresis was carried out in the dark at 50 V for 15 minutes and subsequently at 150 V until the bromophenol dye front reached the lower limit of the gel. Fluorescence images of resolved proteins were acquired by using the Gel Doc XR system (Bio-Rad). Subsequently, gels were stained with Coomassie Brilliant Blue G250 (CBB) and acquired by using GS-800 calibrated densitometer (Bio-Rad, Hercules, USA) at 63 *μ*m resolution. Gel images were analysed using Quantity One 4.6.3 software (Bio-Rad, Hercules, USA). HSA-fluorescence intensity data were normalized for HSA content.

Precision tests were performed as follows: intra-assay CV was evaluated by measuring fluorescent band intensity/*μ*g_HSA_ in the same sample, independently prepared ten times and loaded in the same gel, while inter-assay CV was determined by carrying out the measure on ten consecutive days.

### 2.5. HSA-Bound LMW Thiols Analysis

Levels of Cys^34^-bound LMW thiols were evaluated as described previously [36]. Briefly, circulating and plaque-resident HSA were resolved by nonreducing SDS-PAGE. Then, HSA bands were excised from the gel, destained and LMW thiols extracted by incubating dried bands with 1% tri-n-butylphosphine in aqueous solution (10% tri-n-butylphosphine stock solution in N,N-dimethylformamide). After 5-iodoacetamidofluorescein (5-IAF) derivatization, LMW thiols were resolved by using a P/ACE 5510 CE system with 488 nm Argon ion laser (CE-LIF) (Beckman Coulter, Brea, USA).

### 2.6. Statistical Analysis

Differences between circulating human serum albumin and the corresponding plaque-filtered form were evaluated by using the paired Student's *t*-test. Both levels and distribution of LMW thiols in the two forms have been analysed by using Pearson's Product Moment Correlation test.

## 3. Results

**Figure 1:**
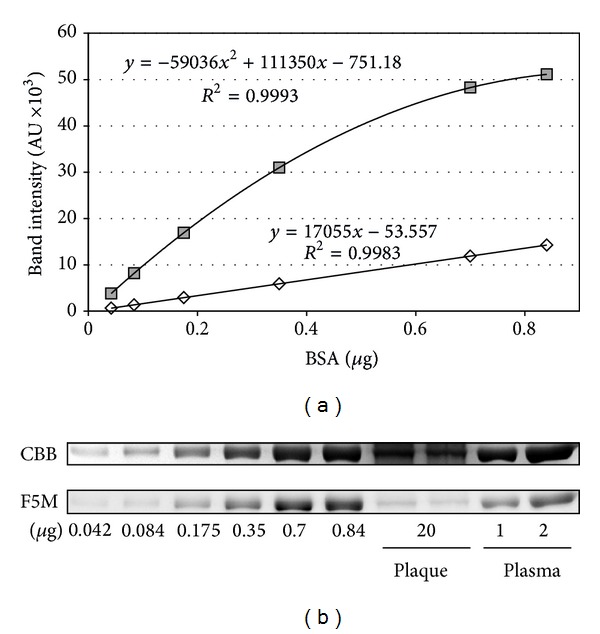
Calibration curves (a) showing a fluorescence linear response of fluorescein-5-maleimide (F5M) labelled BSA over the tested range (0.04–1.0 *μ*g) with a determination coefficient *R* ^2^ = 0.998, and a Coomassie Brilliant Blue G-250 (CBB) second order polynomial response with *R* ^2^ = 0.999 obtained by image analysis on 1D gels (b). AU: arbitrary units; BSA: bovine serum albumin.

**Figure 2:**
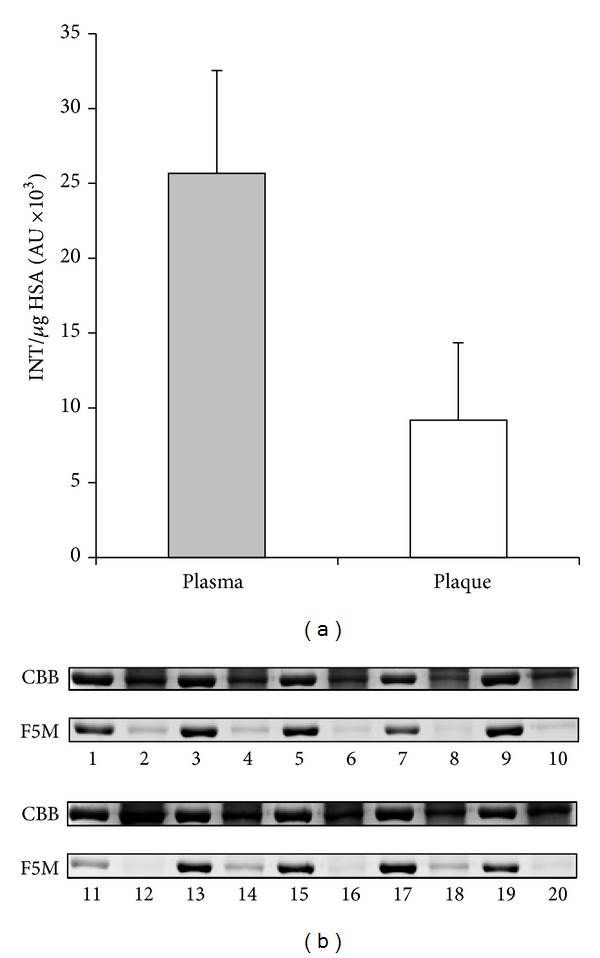
Degree of HSA-Cys^34^ labelling by F5M in plasma and in the corresponding plaque extracts expressed as fluorescent band intensity normalized for *μ*g of HSA (a) obtained by image analysis of 1D gels. Circulating HSA (lanes 1, 3,…) and the corresponding plaque-filtered form (lanes 2, 4,…) from 10 representative patients are reported (b). CBB: Coomassie Brilliant Blue; F5M: fluorescein-5 maleimide.

Preliminarily, we set up calibration curves and performed precision tests on the adopted method for evaluating HSA Cys^34^ total oxidation (Figure 1). Fluorescein-5-maleimide is a reagent effective for labelling free sulfhydryl-containing molecules since, at pH 7, the maleimide group is ~1,000 times more reactive toward a free sulfhydryl than to an amine [38]. Intra- and inter-assay CVs were 2.48% and 4.40%, respectively. Analyses evidenced deep differences between the circulating form of HSA and the corresponding filtered in plaque, the latter being about 2.8-fold less fluorescent with a *P* value < 0.001 (Figure 2).

**Figure 3:**
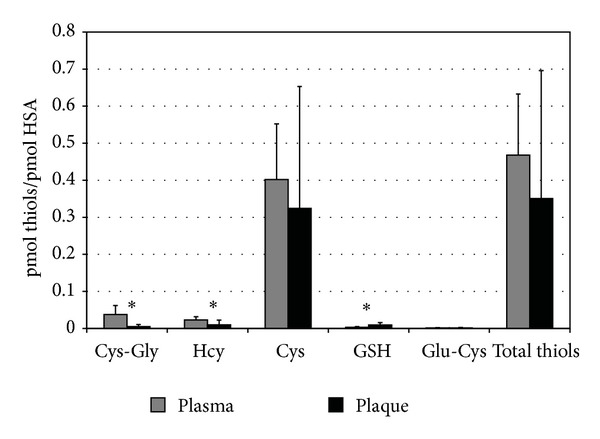
Levels of LMW thiols extracted from both circulating and plaque-filtered HSA, expressed as pmoles per pmoles of albumin, obtained by CE-LIF analysis. *Significant differences between the two HSA forms (*P* < 0.001). Cys-Gly: cysteine-glycine. HCy: homocysteine. Cys: cysteine. GSH: glutathione. Glu-Cys: glutamyl-cysteine.

**Figure 4:**
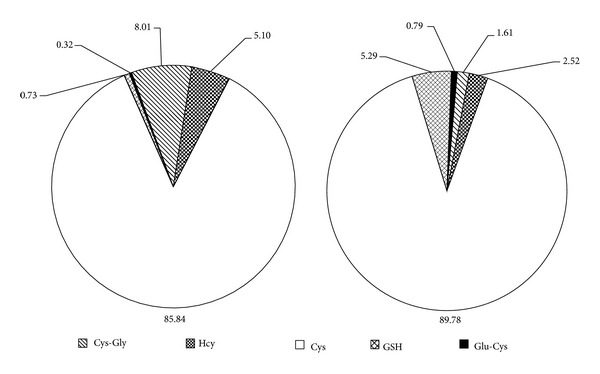
Pattern of S-thiolation of circulating (a) and filtered HSA (b).

CE-LIF analyses evidenced no differences in total levels of HSA-bound LMW thiols (35% versus 47% of thiolation, *P* = 0.097) but a significant reduction of Cys-Gly (~7-fold) and Hcy (~2-fold) as well as an increase of GSH (~2.8-fold) in plaque-filtered HSA compared to the circulating form (Table 1 and Figure 3) that reflect distinct patterns of thiolation (Table 2 and Figure 4). Overall, results on Cys^34^ thiolation highlight that, once filtered into the plaque environment, HSA releases 15.8 ± 10.9 and 32.4 ± 24.9 pmoL/nmoL HSA of HCy and Cys-Gly, respectively (corresponding to 16.2 ± 11.2 and 32.8 ± 23.9 nmoL/g extracted proteins for HCy and Cys-Gly, resp.), which is noteworthy considering the high HSA levels in plaque extracts (971.7 ± 536.9 nmoL/g extracted proteins). Pearson's correlation tests showed no correlation between LMW thiols bound to circulating HSA and the corresponding plaque-filtered form (Table 3).

## 4. Discussion

It is generally held that atherosclerotic plaques are characterized by a proinflammatory and prooxidant environment [39]. Previously, by applying proteomics to the study of carotid plaque vulnerability, we identified a panel of proteins differentially expressed in stable/unstable lesions, with prooxidant and proinflammatory potentials, according to our current understanding of the molecular basis of the atherosclerotic process [34]. Furthermore, the study evidenced that about 70% of extractable proteins from plaques were of plasma origin, with albumin being the most represented [34]. Recently, we focused on some protein oxidative modifications, which might occur in the plaque environment, observing a higher degree of protein sulfhydryl oxidation of both plasma-derived and topically expressed proteins in unstable plaques, partly due to higher levels of S-thiolation [35]. *In situ* oxidative events may have important functional consequences on protein metabolic fate as well as on their bioactivity and antigenic properties. Therefore, in this study, we evaluated albumin Cys^34^ oxidation/thiolation that could follow its subendothelial infiltration in atherosclerotic plaque.

The degree of Cys^34^ oxidation was evaluated by fluorescein-5-maleimide labelling of plasma and plaque extracts. Samples were resolved by nonreducing SDS-PAGE and analysed for fluorescent band intensity after normalization for HSA quantity. The Cys^34^ residue of plaque-filtered HSA was almost 3 times more oxidized with respect to the corresponding circulating form, indicating that the latter, once filtered in plaque, is subjected to Cys^34^ oxidative modifications, probably due to the strong prooxidant environment.

Degree and pattern of protein S-thiolation are the result of both reactivity and levels of LMW-thiols and of protein-SH groups microenvironment. S-Thiolation of circulating albumin by LMW thiols is the most prevalent Cys^34^ oxidative modification. Although the proinflammatory mechanisms mediated by LMW thiols are not yet completely understood, one interesting hypothesis suggests that albumin could be an homocysteine vehicle inside the cells where it could exert its noxious effects. In particular, after proteolysis in lysosomes, both Hcy and the other LMW thiols could be released into the cytosol, where they may alter the intracellular redox potential or modify intracellular proteins resulting in cellular dysfunction [25].

We evaluated albumin Cys^34^ thiolation in plasma samples and in the corresponding plaque extracts by a new approach consisting of a preanalytical HSA purification by nonreducing SDS-PAGE, *in gel* extraction of LMW thiols and analysis by CE-LIF [36]. Although no differences in total HSA-bound LMW thiols levels between the circulating and filtered forms were found, the obtained results evidenced pattern of thiolation specific for the vascular compartment in which HSA resides. In confirmation of these findings, Pearson's test indicated no correlation between levels of LMW thiols bound to the two forms. Interestingly, GSH was significantly higher while both Cys-Gly and Hcy were lower in plaque-filtered HSA. Our data demonstrates, for the first time, that, once filtered, albumin releases significant amounts of Hcy and Cys-Gly in the plaque environment corresponding to 16.2 ± 11.2 and 32.8 ± 23.9 nmoL/g extracted proteins, respectively. Compared to our previous data on total and protein-bound intraplaque LMW thiols [33], such levels represent the bulk of free Hcy and Cys-Gly inside the plaque environment. The different equilibrium in the LMW thiols bound to filtered albumin, with respect to the circulating form, could be partly explained by the high intraplaque GSH levels [33]. We hypothesize that, after being filtered into the carotid subendothelial space, albumin is subjected to Cys^34^-glutathionylation leading to the release of both Hcy and Cys-Gly in the plaque environment. The high intraplaque GSH levels are probably due to cell lysis during apoptotic and/or necrotic events, as suggested by the positive correlation between haemoglobin and GSH levels previously described in carotid plaque extracts [33]. In this regard, haemoglobin represented about 2.6% of total extracted proteins [34], suggesting the relevance of red blood cell lysis in the elevated levels of intraplaque GSH. We have previously demonstrated that circulating LDL apolipoprotein B-100 is able to bind all plasma thiols [30–32]. After infiltration in subendothelial space, oxidative events could lead to proatherogenic LDL isoforms more susceptible to internalization by macrophages. Together with our previous report [33], the present results highlight *in situ* oxidative modifications of plaque extractable protein sulfhydryl groups that could play noteworthy roles in atherosclerotic plaque development and deserve further investigations. Since no differences in the degree of total Cys^34^ thiolation between the two forms of albumin have been detected, the higher degree of oxidation observed could be ascribed to other oxidative modifications driven by ROS, RNS, and reactive electrophilic aldehydes [10–12].

## 5. Conclusions

By comparing circulating and plaque-filtered HSA, we evidenced that the prooxidant environment present in atherosclerotic plaque could modify filtered proteins also by protein-SH group oxidation, probably contributing to plaque progression. Moreover, the results showed patterns of HSA thiolation specific for the filtered form and demonstrated, for the first time, that albumin is a homocysteine and cysteinylglycine vehicle inside the plaque environment. In this respect, the contribution of GSH to the intra-plaque protein-bound LMW thiols equilibrium seems to be of particular importance. For the first time, such a modification in a plasma protein largely filtered in carotid plaque has been described.

## Figures and Tables

**Table 1:** Levels of HSA-bound LMW thiols in plasma and plaque assayed by CE-LIF analysis.

HSA-bound thiols	Plasma	Plaque	Plaque versus plasma
	(pmol/nmol HSA)	(pmol/nmol HSA)	*P* value*
Cys-Gly	37.5 ± 24.5	5.1 ± 5.6	**<0.001**
Hcy	23.2 ± 8.7	9.8 ± 12.8	**<0.001**
Cys	402.2 ± 150.1	324.2 ± 329.0	0.227
GSH	3.4 ± 1.9	9.6 ± 6.5	**<0.001**
Glu-Cys	1.5 ± 0.9	1.8 ± 1.2	0.347
TOTAL Thiol	468.8 ± 165.2	351.1 ± 345.6	0.097

Values are mean ± SD.

Significant differences are reported in bold (*P* < 0.05).

^∗^Paired Student's *t*-test.

**Table 2:** Distribution of HSA-bound LMW thiols in plasma and plaque.

HSA-bound thiols	Plasma (%)	Plaque (%)	Plaque versus plasma *P* value*
Cys-Gly	8.01 ± 4.22	1.61 ± 1.26	**<0.001**
Hcy	5.10 ± 1.69	2.52 ± 1.07	**<0.001**
Cys	85.84 ± 5.15	89.78 ± 5.80	**<0.001**
GSH	0.73 ± 0.33	5.29 ± 5.55	**<0.001**
Glu-Cys	0.32 ± 0.15	0.79 ± 0.54	**<0.001**

Values are mean ± SD.

Significant differences are reported in bold (*P* < 0.05).

^∗^Paired Student's *t*-test.

**Table 3:** Pearson's correlations between HSA-bound LMW-thiols levels in plasma and in plaque.

HSA-bound thiols	Correlation coefficient	*P* value
Cys-Gly	0.049	0.812
Hcy	−0.144	0.482
Cys	0.275	0.174
GSH	0.269	0.183
Glu-Cys	0.005	0.980

## References

[1] Quinlan GJ, Martin GS, Evans TW (2005). Albumin: biochemical properties and therapeutic potential. *Hepatology*.

[2] Peters T (1996). *All about Albumin: Biochemistry, Genetics, and Medical Applications*.

[3] Gryzunov YA, Arroyo A, Vigne J-L (2003). Binding of fatty acids facilitates oxidation of cysteine-34 and converts copper-albumin complexes from antioxidants to prooxidants. *Archives of Biochemistry and Biophysics*.

[4] Iglesias J, Abernethy VE, Wang Z, Lieberthal W, Koh JS, Levine JS (1999). Albumin is a major serum survival factor for renal tubular cells and macrophages through scavenging of ROS. *The American Journal of Physiology*.

[5] Moran EC, Kamiguti AS, Cawley JC, Pettitt AR (2002). Cytoprotective antioxidant activity of serum albumin and autocrine catalase in chronic lymphocytic leukaemia. *British Journal of Haematology*.

[6] Kouoh F, Gressier B, Luyckx M (1999). Antioxidant properties of albumin: effect on oxidative metabolism of human neutrophil granulocytes. *Farmaco*.

[7] Bolitho C, Bayl P, Hou JY (2007). The anti-apoptotic activity of albumin for endothelium is mediated by a partially cryptic protein domain and reduced by inhibitors of G-coupled protein and PI-3 kinase, but is independent of radical scavenging or bound lipid. *Journal of Vascular Research*.

[8] Turell L, Radi R, Alvarez B (2013). The thiol pool in human plasma: the central contribution of albumin to redox processes. *Free Radical Biology and Medicine*.

[9] Ogasawara Y, Mukai Y, Togawa T, Suzuki T, Tanabe S, Ishii K (2007). Determination of plasma thiol bound to albumin using affinity chromatography and high-performance liquid chromatography with fluorescence detection: ratio of cysteinyl albumin as a possible biomarker of oxidative stress. *Journal of Chromatography B*.

[10] Stamler JS, Jaraki O, Osborne J (1992). Nitric oxide circulates in mammalian plasma primarily as an S-nitroso adduct of serum albumin. *Proceedings of the National Academy of Sciences of the United States of America*.

[11] Carballal S, Alvarez B, Turell L, Botti H, Freeman BA, Radi R (2007). Sulfenic acid in human serum albumin. *Amino Acids*.

[12] Aldini G, Vistoli G, Regazzoni L (2008). Albumin is the main nucleophilic target of human plasma: a protective role against pro-atherogenic electrophilic reactive carbonyl species?. *Chemical Research in Toxicology*.

[13] Dröge W (2002). The plasma redox state and ageing. *Ageing Research Reviews*.

[14] Soejima A, Matsuzawa N, Hayashi T (2004). Alteration of redox state of human serum albumin before and after hemodialysis. *Blood Purification*.

[15] Watanabe A, Matsuzaki S, Moriwaki H, Suzuki K, Nishiguchi S (2004). Problems in serum albumin measurement and clinical significance of albumin microheterogeneity in cirrhotics. *Nutrition*.

[16] Suzuki E, Yasuda K, Takeda N (1992). Increased oxidized form of human serum albumin in patients with diabetes mellitus. *Diabetes Research and Clinical Practice*.

[17] Kadota K, Yui Y, Hattori R, Murohara Y, Kawai C (1991). Decreased sulfhydryl groups of serum albumin in coronary artery disease. *Japanese Circulation Journal*.

[18] Djoussé L, Rothman KJ, Cupples LA, Levy D, Ellison RC (2002). Serum albumin and risk of myocardial infarction and all-cause mortality in the framingham offspring study. *Circulation*.

[19] Schillinger M, Exner M, Mlekusch W (2004). Serum albumin predicts cardiac adverse events in patients with advanced atherosclerosis—interrelation with traditional cardiovascular risk factors. *Thrombosis and Haemostasis*.

[20] Austin RC, Lentz SR, Werstuck GH (2004). Role of hyperhomocysteinemia in endothelial dysfunction and atherothrombotic disease. *Cell Death & Differentiation*.

[21] Tsai J-C, Perrella MA, Yoshizumi M (1994). Promotion of vascular smooth muscle cell growth by homocysteine: a link to atherosclerosis. *Proceedings of the National Academy of Sciences of the United States of America*.

[22] Bescond A, Augier T, Chareyre C, Garçon D, Hornebeck W, Charpiot P (1999). Influence of homocysteine on matrix metalloproteinase-2: activation and activity. *Biochemical and Biophysical Research Communications*.

[23] Tyagi SC, Smiley LM, Mujumdar VS, Clonts B, Parker JL (1998). Reduction-oxidation (Redox) and vascular tissue level of homocyst(e)ine in human coronary atherosclerotic lesions and role in extracellular matrix remodeling and vascular tone. *Molecular and Cellular Biochemistry*.

[24] Okamoto T, Akaike T, Sawa T, Miyamoto Y, van der Vliet A, Maeda H (2001). Activation of matrix metalloproteinases by peroxynitrite-induced protein S-glutathiolation via disulfide S-oxide formation. *The Journal of Biological Chemistry*.

[25] Sengupta S, Chen H, Togawa T (2001). Albumin thiolate anion is an intermediate in the formation of albumin-S-S-homocysteine. *The Journal of Biological Chemistry*.

[26] Schnitzer JE, Oh P (1994). Albondin-mediated capillary permeability to albumin. Differential role of receptors in endothelial transcytosis and endocytosis of native and modified albumins. *The Journal of Biological Chemistry*.

[27] Tiruppathi C, Finnegan A, Malik AB (1996). Isolation and characterization of a cell surface albumin-binding protein from vascular endothelial cells. *Proceedings of the National Academy of Sciences of the United States of America*.

[28] Schnitzer JE, Sung A, Horvat R, Bravo J (1992). Preferential interaction of albumin-binding proteins, gp30 and gp18, with conformationally modified albumins. Presence in many cells and tissues with a possible role in catabolism. *The Journal of Biological Chemistry*.

[29] Schnitzer JE, Bravo J (1993). High affinity binding, endocytosis, and degradation of conformationally modified albumins. Potential role of gp30 and gp18 as novel scavenger receptors. *The Journal of Biological Chemistry*.

[30] Zinellu A, Sotgia S, Deiana L, Carru C (2005). Quantification of thiol-containing amino acids linked by disulfides to LDL. *Clinical Chemistry*.

[31] Zinellu A, Sotgia S, Zinellu E (2006). Distribution of low-density lipoprotein-bound low-molecular-weight thiols: a new analytical approach. *Electrophoresis*.

[32] Zinellu A, Zinellu E, Sotgia S (2006). Factors affecting S-homocysteinylation of LDL apoprotein B. *Clinical Chemistry*.

[33] Zinellu A, Lepedda A, Sotgia S (2009). Evaluation of low molecular mass thiols content in carotid atherosclerotic plaques. *Clinical Biochemistry*.

[34] Lepedda AJ, Cigliano A, Cherchi GM (2009). A proteomic approach to differentiate histologically classified stable and unstable plaques from human carotid arteries. *Atherosclerosis*.

[35] Lepedda AJ, Zinellu A, Nieddu G (2013). Protein sulfhydryl group oxidation and mixed-disulfide modifications in stable and unstable human carotid plaques. *Oxidative Medicine and Cellular Longevity*.

[36] Zinellu A, Lepedda A, Sotgia S (2010). Albumin-bound low molecular weight thiols analysis in plasma and carotid plaques by CE. *Journal of Separation Science*.

[37] Mastro R, Hall M (1999). Protein delipidation and precipitation by tri-n-butylphosphate, acetone, and methanol treatment for isoelectric focusing and two-dimensional gel electrophoresis. *Analytical Biochemistry*.

[38] Bigelow DJ, Inesi G (1991). Frequency-domain fluorescence spectroscopy resolves the location of maleimide-directed spectroscopic probes within the tertiary structure of the Ca-ATPase of sarcoplasmic reticulum. *Biochemistry*.

[39] Libby P (2012). Inflammation in atherosclerosis. *Arteriosclerosis, Thrombosis, and Vascular Biology*.

